# Facilitators and barriers to the clinical administration of herbal medicine in Ghana: a qualitative study

**DOI:** 10.1186/s12906-021-03334-x

**Published:** 2021-06-30

**Authors:** Comfort Asare, Lydia Aziato, Daniel Boamah

**Affiliations:** 1 School of Nursing, Wisconsin International University College. Ghana, P.O. Box KS 5903, Adum Kumasi, Ghana; 2 Department of Adult Health, School of Nursing, University of Ghana, Legon Accra, Ghana; 3 Centre for Plant Medicine Research, Mampong Akuapem,, Ghana

**Keywords:** Qualitative, Complementary and alternative medicine (CAM), Ghana, Herbal medicine, Facilitators, Barriers, Nurses

## Abstract

**Background:**

Herbal medicine administration in conventional health care services is gaining popularity lately. Much has not been documented on the perceived enhancers and challenges to herbal medicine administration at the hospital. The study sought to explore the facilitators and barriers to the clinical administration of herbal medicine in Ghana.

**Method:**

Qualitative descriptive exploratory design was employed. Fourteen participants among the consented and purposively sampled nurses were interviewed. Data was transcribed and analysed using content analysis.

**Results:**

The participants disclosed that facilitators to the clinical administration of herbal medicine include doctors’ prescription, affordability of herbal medications by patients, patients’ willingness to use herbal medicine and availability of herbal medicine. Barriers to the clinical administration of herbal medicine were inadequate knowledge on herbal medicine, lack of publicity, unclear integration, lack of collaboration and policies on herbal medicine administration at the hospital. Other barriers were negative mindset of patients and lack of national health insurance scheme (NHIS) coverage.

**Conclusion:**

Clinical administration of herbal medicine is faced with an array of challenges. Doctor’s prescription, nursing education on herbal medicine and NHIS coverage of herbal medicine are imperative to improve herbal medicine administration in hospitals.

**Plain English summary:**

Herbal medicine addition into mainstream health care services is surging high in many countries. This study aimed at finding out what nurses consider as the issues that make it easy or difficult to serve herbal medicine in the hospital.

Qualitative method was employed, in-depth face-to-face interviews were conducted and data collected was typed verbatim. The typed data was content analysed and findings supported with the nurses’ statements.

The findings of the study showed that facilitators to the clinical use of herbal medicine include doctors’ prescription, affordability of the herbal drug, patient’s willingness to use the herbal medication, patient’s belief about herbal medicine and availability of herbal medicine. Challenges to the clinical use of herbal medicine disclosed were lack of knowledge on herbal medicine, lack of publicity, unclear integration, lack of collaboration between health professionals and herbal medicine providers. Other barriers include negative mindset of patients and lack of national health insurance (NHIS) coverage.

The researchers came to a consensus that nurses need further training on herbal medicine to enhance herbal medicine use at the hospital. Health professionals need to collaborate with herbal medicine service providers and NHIS must be reviewed to cover herbal medications.

## Background

The beneficial role of plants has been acknowledged over the years [1]. Herbal medicines, extracted from plants are known to treat illness across different cultures in the world [1, 2] and it is also a type complementary and alternative medicine (CAM) [3]. Traditional herbal medicine is a medications naturally made from plants with little or without industrial effect [2]. In Germany, continuous use of herbal medicine is predominant among 86.7% of the general population [4]. An average of 58.2% of the population in Ethiopia and Nigeria are reported to use traditional complementary and alternative medicine [5]. In Ghana traditional medicine use is prevalent among 70% of the population [6, 7].

Herbal medicine has been noted for the management of several conditions in Africa [8–12]. For instance it is used in the treatment of hypertension in South Africa and Tanzania [10, 11]; conditions of the respiratory tract, gastrointestinal tract and general bodily pain [10]. Some students of the University of Nigeria used herbal medicine to manage malaria and typhoid fever [12]. In Ghana, herbal medicine is used in the management of cancer and the various phases of pregnancy [8, 9].

A number of studies have reported nurses clinical administration of some CAM therapies including herbal medicine [13–16]. According to [13] some nurses have used CAM therapies in managing pains in patients with cancer in different parts of the world. In Norway, some nurses managed challenging behaviours of patients with dementia by using herbal medicine. They administered a dose of lavender, a form of aroma therapy on the palm and neck of the patients after which they became calm [16]. Some nurses in Israel changed the way they spoke with their patients because they were guided by the training they had received on guided imagery. This ensured lower levels of anxiety in their patients [17]. In Ghana some nurses and midwives who personally experienced positive results with herbal medicine use recommended the therapy to their patients including pregnant women [18, 19]. A few studies have shown that nurses who experienced favorable outcome after administering CAM to their patients yearned to work with it daily since they relished the ability to serve such therapies [17, 20]. However in Iran, most of the nurses (57.3%) involved in a study by [14] did not apply CAM in the clinical practice. The few who administered CAM on the ward mostly practiced massage, prayer and herbal medicine.

Mainstream health care administration of herbal medicine has been met with factors that either expedites or challenges the process [13, 21, 22]. Some of the overarching factors proposed to be facilitating the utilization of CAM therapies include availability of herbal medications and belief in positive benefits of CAM among nurses, physicians and patients [13, 21, 23–26]. Perceived barriers reported comprised lack of prescription by physicians, lack of collaboration between health professionals and CAM providers, high cost of CAM, lower level of knowledge on CAM among nurses and other health professionals [13, 22, 24, 26–28]. Studies at the Brim South District and the Kumasi South Hospital of Ghana also reported inadequate knowledge and ignorance of protocols regarding herbal medicine administration in hospitals among health professionals [18, 19].

In Ghana, a Traditional and Alternative Medicine Directorate has been established since 1991 as part of the Ministry of Health. The directorate is obliged with advocating and advancing traditional medicines particularly herbal medicines [29]. The traditional medicine Act 2000 was subsequently enacted to control herbal and other traditional medicines in 1992. In compliance with the Act, scientifically proven herbal medications are produced by the Centre for Plant Medicine Research in addition to the production of trained herbal doctors from a public university [29]. The Traditional Medicine Practice Council was also established in 2010 to regulate and license hospitals and practitioners of CAM in Ghana [30]. In 2012 herbal medicine was merged into mainstream hospital care at fifteen (15) regional hospitals [29, 31]. The medications are prescribed by the trained herbal doctors from the University [30]. Since serving of medication is one of the essential functions of nurses in health facilities [32, 33], they are likely to administer herbal drugs in institutions with herbal units.

Previous studies have shown that the success of CAM integration into hospital care is largely linked with the involvement of nurses [15, 34]. However what nurses recognize to either enhance or hinder clinical administration of herbal medicine in Ghana is not largely documented. This study sought to explore what nurses consider as the facilitators and barriers to the clinical administration of herbal medicine.

## Methodology

### Design

An exploratory descriptive qualitative design was adapted in this study. The design helped to gain detailed understanding of what the nurses’ thought are the facilitators and barriers to the clinical administration of herbal medicine [35, 36] since much is not known about this in Ghana [37]. The design allowed the nurses to fully describe the facilitators and barriers to herbal medicine administration at the hospital.

### Setting

The study setting was a public hospital in Accra Ghana that runs both conventional and nonconventional services with one hundred and seven (107) bed capacity. The facility runs an out-patient herbal service for individuals who seek herbal drug management. The herbal unit has two herbal doctors and a nurse. Other categories of health professionals at the hospital include doctors, nurses, midwives, sonographers, dentist, pharmacist, herbal doctors and laboratory staff. The participants had diverse backgrounds such as general nursing, midwifery and enrolled nursing and were drawn from different wards of the hospital.

### Target population and sampling technique

The target population was registered nurses and midwives with at least 2 years working experience at the hospital. Newly qualified nurses on rotation were excluded as well as those who have not worked continuously for 2 years at the hospital. Among the participants who consented, met the inclusion criteria and sampled purposively, fourteen (14) were interviewed.

## Method of data collection

After permission was granted by the hospital authorities, the nurses were approached after a formal introduction of the researcher by a staff at the nursing administration. The purpose, objectives and inclusion criteria were explained to them and those who volunteered were provided with consent forms to obtain their written consent. Participants were contacted subsequently to schedule for an interview on a day, time and venue suitable to them. Participants were engaged in individual face-to-face interviews which were all conducted in English language by the first author; who was trained and supervised by the second author – a qualitative research expert.

A semi-structured interview guide with open ended questions based on the objectives of the study was used in data collection. The questions permitted profound probing of emerging themes. For example, participants were asked: *What will make it easy for you to serve herbal medicine? What will you require for effective herbal medicine administration? Tell me some of the challenges you have with herbal medicine administration.* Participants freely expressed themselves without any interference during the interviews. Each interview took between 45munites to one (1) hour. By the fourteenth participant data was saturated since no new idea was obtained from the participants. All interviews were audio recorded and transcribed verbatim. Each participant was refreshed with a bottle of malt drink and a piece of meat pie after their individual interviews.

### Data management and analysis

Data was analysed concurrently and it followed the process of content analysis as suggested by [38, 39]. Data was transcribed by the first author and an assistant The data analysis was done manually by the first and second authors. The transcripts were read several times to fully comprehend the responses provided by the participants. The transcribed data was read line after line and codes were assigned. Codes of similar meanings were further regrouped to form subthemes. Similar subthemes were regrouped and refined to generate themes. Field notes were kept during the interviews to record the tone of the nurses’ speeches and their nonverbal responses such as their gestures and other body languages. This helped the researchers to understand and analyse the data. The authors further discussed the themes to ensure that the nurses were sincerely presented. Direct verbatim quotes from the nurses were used to support the findings.

### Ethical consideration

Ethical approval was given by the Institutional Review Board of the Noguchi Memorial Institute of Medical Research of the University of Ghana (NMIMR-IRB CPN 103/13–14 amend. 2015). All participants consented to the study and were informed they could withdraw at any time without any consequence. Permission was sought from the participants to record the interviews and future publication. Data presented has no identifying information. To ensure confidentiality, each participant was given an identification code such as NI, N2, N3 etc. depending on the order in which participants were enrolled. All methods were carried out in accordance with relevant guidelines and regulations.

### Trustworthiness of the study

Trustworthiness was enhanced through a number of processes. Concurrent analysis of data ensured probing of emerging themes in subsequent interviews in order to gain full understanding of the themes. The same interview guide was used for all the nurses. Only nurses with 2 years or more working experience at the hospital were recruited. Detailed field notes were kept which allowed for verification and understanding responses. Direct verbatim quotes of the nurses were used to support the findings.

## Results

### Characteristics of study participants

The participants were fourteen (14) which comprised eleven (11) nurses, two (2) midwives and an enrolled nurse. They were all female, Christians and had work 2 years or more. The participants were drawn from different units such as the herbal, paediatric, male, female, maternity, outpatient department and anti-retroviral units. They were aged between twenty-five (25) to sixty (60) and made up of diploma, bachelor of nursing, midwifery and enrolled nursing certificate holders. All participants resided in the Accra Metropolis. Table 1 presents details of participants’ demographic characteristics.

**Table 1:** Participant’s demographic background

Variable	Response	Number
Specialty	General nurses	11
	Midwives	2
	Enrolled nurses	1
Gender	Female	14
	Male	0
Religion	Christianity	14
	Islam	0
	Others	0
Ward /Unit	ART	2
	OPD	2
	Medical ward	2
	Surgical ward	3
	Paediatric ward	2
	Herbal Unit	1
	Maternity	2
Age	18–28	4
	29–39	6
	40–50	1
	51–60	3
Academic qualification	Enrolled Nursing Certificate	1
	Diploma	10
	Midwifery Certificate	2
	BSc Nursing	1
Number of years of service	2–11 years	10
	12–21 years	2
	22–31 years	0
	32–41 years	2

The findings were described on two themes thus, facilitators of clinical administration of herbal medicine and barriers to clinical administration of herbal medicine. The themes had corresponding subthemes. The themes and subthemes are presented in Table 2.

**Table 2:** Themes and subthemes

Themes	Subthemes
1. Facilitators of clinical administration of herbal medicine	1. Facilitating factors
2. Barriers to clinical administration of herbal medicine	1. Knowledge on herbal medicine 2. Publicity of herbal unit 3. Integration of herbal medicine into hospital care 4. Patient’s mind set 5. National health insurance coverage.

### Facilitators of clinical administration of herbal medicine

This theme describes what the nurses thought would facilitate administration of herbal medicine at the hospital. The nurses expressed a subtheme that related to facilitating factors which has been described with supporting quotes from the participants.

### Facilitating factors

The nurses believed they were not responsible for prescribing medications. However, based on their knowledge on pharmacology they make suggestions to doctors for adjustments of medications where necessary. The decision to serve herbal medicine in the hospital was dependent on doctors prescribing it. The nurses were ready to serve if only the doctors prescribe.

*“As nurses we do not prescribe medications. On few occasions because of our knowledge in pharmacology we are able to suggest to doctors to adjust the dosages of some medications. It is not solely our decision to serve herbal medicine. It depends on doctors prescribing it; if the doctors prescribe, I will serve”* **N1**

*“Once the doctor writes it, I am okay to serve.”* **N5**

The nurses were expecting the prescription to be written by the doctors and stamped in the patient’s folder to facilitate administration of herbal medicine to patients.

*“If the prescription comes in the doctor’s handwriting, the doctor’s stamp and written in the patient’s folder; I will serve it”* **N4**

Some participants stressed the need for nurses to serve only prescribed herbal medications to obtain the support of the hospital authorities should the unexpected happen. The nurses expected their colleagues to be very clear with the instructions regarding the prescriptions.

*“We only administer prescribed drugs so if the drug is not prescribed and you administer it and maybe a problem erupts, I don’t think the hospital will back you.”* **N12**

*“Nurses should be clear with the instructions concerning the prescribed herbal medication. If any nurse is not clear with the instructions, he/she should question the doctor who prescribed the medication.”* **N8**

Nurses thought that if only patients could afford the herbal medication it will be easy for nurses to serve.

*“If patients are able to afford the herbal medicine we will serve. When the doctor prescribes and they buy it then we have no business than to serve it”* **N5**

*“If the doctor prescribes the herbal medicine and the patient is ready to pay, we will serve. “*N**6**

According to the nurses, the patient’s willingness and beliefs towards herbal medicine was also going to make it easy to serve the herbal medication. A patient with positive belief in herbal medicine surely take the herbal medication.

*“Patient’s willingness to take it too will also count. I believe that the patient’s willingness to take it will make it easier.”* **N4**

*“It depends on the individual’s beliefs. There are some people that have confidence in herbal medicine no matter what. So, such people if you give them, it will work.”* **N5**

Some of the nurses also stated that the availability of the drugs was very important in making it easy for them to serve herbal medicine. They were interested in the drug being continuously available.

*“The availability of the drugs will really matter. Sometimes you will want the drugs to be available so that you can serve but the suppliers will not bring them. So, if it will be made continuously available it will be easier.”* **N4**

A few participants thought integrating herbal drugs into conventional pharmacy unit will improve patronage and recommendation by those who experience a positive outcome.

*“It will help if the herbal medicine is available at the pharmacy. It will even encourage people to use it the more and if it works for them, they will tell others to patronize it.”* **N5**

However, a nurse recommended that once nurses are mandated to serve the herbal medications, it should be made available.

*“I think if it’s going to be legally permitted for nurses to administer herbal medicine in the hospital, then the herbal medication should be made available for patients who opt for it.”* **N11**

### Barriers to clinical use of herbal medicine

This theme describes the factors which were seen as hindrances to the clinical administration of herbal medicine at the hospital. They included inadequate knowledge on herbal medicine, patients with negative mindset towards herbal medicine, unclear integration of herbal medicine into mainstream health care, lack of publicity for herbal unit and lack of national health insurance coverage. The subthemes obtained are described with verbatim quotes from the nurses.

### Knowledge on herbal medicine

Almost all the participants in this study believed they lacked the necessary knowledge on herbal medicine which was perceived as the main hindrance to the clinical use of herbal medicine. The nurses felt uncomfortable to administer herbal preparations in the future regardless of the years they have been in the profession.

*“We don’t know much about herbal medicine so we are not very comfortable to administer it. We don’t have much training and that is our main hindrance.”* **N1**

*“Our knowledge on herbal medicine is not enough; we need more education. Having been in nursing practice for so many years, I don’t think the knowledge I have is enough.”* **N6**

Some of the nurses were not taught herbal medicine at all during their professional training, others studied traditional medicine or herbal medicine for just a semester and it was not considered enough.

*“At school we were not taught to use herbal medicine, we don’t even know the drugs, we don’t know what they do.”* **N8**

*“When we were in school, we were taught traditional medicine for only one semester.”* **N4**

*“You know my training; we did traditional medicines along the line but it wasn’t enough.”* **N12**

The participants were expecting to be abreast with the knowledge on the uses of herbal preparations, types of herbal medicines and the routes of administration.

*“If nurses have proper training on the use of herbal preparations; so that we become abreast with the knowledge on herbal medicine, I will serve.”* **N1**

*“If we are to serve now then we need more education so that we will be knowledgeable on the types and the route of administration.”* **N6**

Participants stated they really needed education on herbal pharmacology because serving of herbal medicines involves human life which could be fatal and nurses needed to be cautious. They expected herbal pharmacology books to be available like that of orthodox medicines.

*“We really need to be educated on herbal pharmacology to delve into critical issues. We are dealing with human beings and in dealing with human life you have to be very careful; every aspect of it is sensitive. The least mistake you do will cost somebody’s life.”* **N4**

*“I think we need more education on herbal medicine. Just as there are books on orthodox medication pharmacology there should be books on herbal pharmacology for us to read so that we can administer.”* **N11**

In addition, participants were interested in the training because they thought it will help them to be knowledgeable in the efficacy of herbal medicine to deliver and be courageous in their service delivery.

*“With time if we have education we can deliver.”* **N6**

*“You have to give us the training so that we know the efficacy of herbal medications and have the courage to serve our patients.”* **N13**

The nurses were sure education on herbal medicine would eradicate every negative feeling of health professionals towards herbal medicine and make them confident to convince patients to use herbal medications.

*“A doctor may not believe in prescribing herbal medicine to patients; a nurse may also not believe in it and will not serve. However, through education their confidence would be built and they will serve it.”* **N4**

*“Well I believe the training and education will eliminate every uncomfortable feeling.”* **N1**

They expected the education to be in the form of workshops or in-service training and not necessarily going back to the classroom to be taught all over again.

*“I think there should be more workshops or training; may be an in-service training for staff before herbal medicines will be used on the ward.”* **N10**

*“I think we can organise workshops instead of going back to the classroom.”* **N11**

Some of the nurses suggested the need for herbal nurses to assist the herbal doctors. They expected the herbal nurses to be knowledgeable than non-herbal nurses. They suggested that specialization in herbal nursing could be a postgraduate course.

*“Since there are herbal doctors trained at the university, they should have their herbal nurses to administer herbal medicine. If they have herbal schools for nurses; for instance, a postgraduate herbal nursing programme, it will be better.”* **N8**

*“I think if herbal doctors train their own herbal nurses it will be better. If you are trained and knowledgeable about it and you are serving patients it is more appropriate. They should have special training school for nurses who are interested”* **N9**

### Publicity of herbal unit

Another barrier identified as a hindrance to the clinical use of herbal medicine was publicity. Some of the nurses thought the hospital was not really promoting herbal medicine; even the public was alleged not to be aware of the existence of the herbal unit at the hospital.

*“I do not think the hospital is promoting it. At the OPD information desk, sometimes they tell patients that we have herbal clinic at the top so those who are interested in herbal medicine should go up stirs. However, they don’t announce it often”* **N5**

*“Most people don’t know that we have a herbal section here; it’s just a few people who know.”* **N11**

*“Even at home people ask of the herbal unit because they don’t know. I am the one who normally tells them we have a herbal unit in the hospital.”* **N13**

A staff at the herbal unit tried promoting a herbal lotion which was very effective for wound dressing but it did not get the necessary acceptance.

*“A staff at the herbal unit came to tell us about asmesol. He said it is a herbal preparation used for dressing of wounds and it was good. I told him to inform the doctors because they prescribe wound dressing lotions but it was not successful.”* **N5**

The location of the herbal unit was also obstructing publicity of the herbal medicine. A participant narrated that the location of the herbal unit made it difficult for the unit to be seen. Patients interested in herbal medicine felt reluctant asking of the unit’s location

*“The problem is where the herbal unit is located; it should have been at a place where it can be identified easily upon entering the hospital. Some people feel shy to ask of where the herbal unit is. However, if the person enters the facility and easily identifies the herbal unit the person will just go there without asking anybody.”* **N13**

The nurses’ reasons why the herbal medicine unit should be advertised include previous lack of approval by hospitals and the possibility of patients perceiving it as a clinical trial.

*“The hospital should promote its administration of herbal medicine because people know that hospitals condemn herbal medicine and discourage patients from using it. If this hospital is now serving herbal medicine then it should be advertised. Otherwise patients may think the hospital is using them for trial and any bad outcome may be linked to the hospital.”* **N7**

### Integration of herbal medicine into hospital care

It was discovered that some nurses at the hospital were not fully clear of herbal medicine integration in the hospital. The herbal unit seemed to be standing alone or separated from the mainstream health care and medical doctors were not prescribing herbal drugs.

*“We don’t know whether herbal medicine has been fully integrated into the healthcare system or they are standing solely on their own. We need to know whether the whole hospital has accepted to merge it with the orthodox medicine. We don’t know whether to tell someone or recommend it to someone. It is not very clear whether there is a full integration or not, I don’t know.”* **N1**

*“It is like there is a barrier; the herbal unit is doing its own thing and we (*main stream healthcare*) are also doing our own thing. We are administering orthodox medicine and they are also giving their herbal medicine.”* **N8**

The nurses expressed that there was the need for all staff to collaborate; they needed to work as a team and tolerate each other to enhance herbal medicine usage. Every profession was to be considered equally important so that the staff could testify about herbal medicine.

*“We should work together; we shouldn’t look down upon each other. We should come to a consensus as to what we actually want to do. If we are initiating herbal medicine into the facility, then we should come together so that when the drug works, we would testify about it.”* **N2**

*“There should be a collaboration; if a patient wants to switch from orthodox medicine to herbal or herbal medicine to orthodox medicine, it should not create tension between herbal doctors and medical doctors. There should not be any disparity; likewise nurses and all other staff whether at the herbal unit or the mainstream healthcare should collaborate.”* **N10**

The majority of the nurses were not aware of policies or protocols that empowered them to serve herbal medicine in the hospital. Legally, the nurses felt they were not backed by law to serve herbal medicine.

*“If there are protocols then I don’t know because I have not seen one. Usually if there is anything like that, they will circulate it to all units and departments but I have not seen any here yet so we don’t have the policies.”* **N7**

*“Of cause policies or protocols to cover nurses; if Ghana health service agrees to the administration or integration of herbal medicine to the normal system* (orthodox healthcare)*, the law should protect us, we should be backed by law before we start serving because there are no policies in place protecting us.”* **N8**

### Patient’s mindset

The mindset of a patient about herbal medicine was an additional barrier to the clinical use of herbal medicine. According to some nurses though the herbal drug may be prescribed, a patient may decide not to take the drug and the nurse cannot coerce him or her to do otherwise.

*“The patient can tell you he or she will not take the medication and it’s a challenge.”* **N2**

*“A doctor may prescribe the herbal medicine for a patient but then the patient may not be interested in taking the medicine which can also be a challenge because you cannot force the patient.”* **N4**

A participant mentioned that when a patient refuse to take the herbal medicine, nurses must come to the patient’s level and find means to convince him or her. The patient must be made to believe it is safe so that the patient can testify about herbal medicine in the future.

*“When the patient is refusing the herbal medication, you have to come down to the patient’s level and let the patient know what you are giving him/her. Let him know he is in safe hands and that he will be fine.”* **N13**

Some participants expressed that the state of a herbal drug to be served to patients on the ward could raise suspicion in a patient. If the herbal drugs to be served are prepared similar to locally prepared ones at home, then patients would be suspicious and not use it.

*“It will be a big challenge if it happens that we have to boil the herbal medication before we serve patients on the ward. Some people may feel some way, even if it was me and you are bringing something to me in a cup, I will feel uncomfortable. The patient may feel uncomfortable with you pouring herbal medicine into a cup to serve him. Something you have poured at the nurses’ station to the bedside. He or she would not be sure of what he is taking. The patient may think you have putting something bad in the medication.”* **N5**

Since the hospital was treating patients with herbal medicine on OPD basis, a participant thought that some patients may add other unapproved herbal medicines to what has been prescribed for them.

*“A patient going home with prescribed herbal medicine will add some other herbal medicine to make his condition worse.”* **N7**

### National health insurance coverage

Absence of insurance cover for herbal drugs was also an impediment to the clinical use of herbal medicine. National Health Insurance Scheme (NHIS) was not covering herbal drugs prescribed at the hospital and this prevented people from patronizing the unit.

*“Some people want herbal medicine but they don’t have the money to buy it because herbal medicines are not covered by NHIS.”* **N13**

*“The only thing that draws clients away from patronizing the herbal unit is the financial difficulty. The herbal medicines are not covered by NHIS; patients cannot bear the cost; most of them don’t have enough to buy.”* **N14**

A few of the nurses recounted that NHIS covered consultations with the herbal doctors but echoed that if NHIS covers herbal drugs it would be helpful since it covered orthodox medicine.

*“I know for their consultations NHIS covers it but I don’t know whether it covers the medications itself but if it covers the medication as well, I think it will also be a good idea because it covers orthodox medicine.”* **N2**

Some nurses recounted incidents at the hospital where patients came in with emergency conditions without money and relatives. They could not afford their drugs because the drugs were not covered by NHIS and the pharmacy was not serving the drugs for free. This could hinder the administration of herbal medicine.

*“Some patients come with no relative and money; they come in the state of emergency. The doctor would diagnose and prescribe and there will be a problem as to how to get the medicine. The drug is not covered by insurance; the patient may not have money and the pharmacy will not serve without the patient paying.”* **N9**

Some nurses expressed that if NHIS covers herbal medicines it would make it easy for other doctors (medical doctors) to prescribe herbal medicine and prevent patients from buying outside.

*“If herbal medicines are covered by NHIS it will help, it will make it easier for doctors to prescribe if they want to.”* **N1**

*“National Health Insurance covering herbal medicine will be good because people will stop buying outside and come to the hospital.”* **N7**

## Discussion

The current study explored the facilitators and barriers to the clinical administration of herbal medicine. The nurses mentioned that doctors’ prescription will make it easy to administer herbal medicine in the future [13, 40]. However the study by [40] was concerned with nurses serving orthodox medication. Participants in the present study were optimistic about them not having the sole right to serve herbal medication [40]. This could be attributed to the professional background of the majority of the participants which currently does not permit them to prescribe medicatons in Ghana. The nurses expectation of doctors’ prescription of herbal medicine was directed towards conventional doctors which could be related to familiarity as well as drug prescription being reported as a responsibility of medical doctors [41]. This might contribute to why the nurses thought they could only make suggestions to the doctors [40]. It is important for policy makers to consider including mainstream healthcare doctors in policies regarding prescription of herbal medicine in hospitals.

The nurses’ recommendation that their colleagues must clearly understand herbal related prescriptions and ensure it is stamped before serving indirectly resonates with [41]. They reported that prescriptions without physician’s signature nullified the physician’s request which hindered serving of medications. Their study was concerned with conventional healthcare systems. A previous study also indicated patients’ refusal of CAM as a treatment option since there was no physician order [42]. This might suggest the need for physician’s order to be very crucial in expediting drug administration in both mainstream and nonconventional systems. Therefore, buttressing the need for conventional doctors’ involvement in prescribing herbal medicine.

Participants also described patients’ ability to afford herbal medications as a factor that could ease serving herbal drugs in the hospital [21, 26, 43]. Nevertheless, other studies submitted affordability as a barrier to the use of herbal medicine at conventional care and private herbal hospitals among nurses and patients [13, 23, 24, 44]. For instance, in Ghana whereas some patients in a private hospital maintained herbal medicine is expensive [24], some health workers at the Kumasi South Hospital which also administers herbal medicines asserted that it was cheap [19]. This might imply that affordability as a facilitator to the clinical administration of herbal medicine could be relative and cannot be generalized. Health workers including nurses should therefore consider affordability critically before administering herbal medications to patients.

The nurses’ assertion that patient’s willingness to take herbal medication will facilitate the clinical administration process is supported in a study by [45] who discovered same in Beijing, China among cancer survivors. However, in Gauteng, South Africa patients demonstrated high level of unwillingness to use herbal medicines again in the management of certain conditions. A few others displayed readiness for use but for specific diseases [10]. This might imply that patient’s willingness to use herbal medicine should be considered as an important factor since herbal medicine administration in the hospital is quiet a new phenomenon in Africa. In addition, the need for patient’s willingness to use herbal medicine as a facilitator might suggest that health workers are not the only people needed to enhance herbal medicine use at the hospital. It is important that healthcare practitioners take cognizance of patient’s willingness to use herbal medications before prescribing or administering.

Another facilitator expressed in this study is patient’s belief regarding herbal medicine which is consistent with a number of studies [4, 13, 21, 28, 46]. The nurses uttered that a patient with a positive belief about herbal medicine will surely take the medication [4, 21, 28, 46]. The previous studies further buttressing the assertion by nurses posited positive beliefs such as the natural nature and beneficial role of CAM as reasons for CAM use [4, 21]. At the Brim South District in the Eastern Region of Ghana, some midwives who had positive belief in the effectiveness of CAM recommended its use among pregnant women attending their facilities [18]. Contrarily, negative belief that traditional medicine use can be fatal also prevented patients from its utilization in Malawi [21]. This might suggest that individuals are unique in their beliefs regarding herbal medicines in Africa. Health authorities must therefore emphasis in their policies the need for patients to initiate the decision to use herbal medicine but must not be without the counsel and guidance of health experts. As submitted by [13] patients are to be allowed to initiate their own choice of treatment.

The nurses reiterated the necessity for herbal medications to be available to facilitate administration in the future which is partly reported in India, Malawi and Ghana [18, 21, 26]. Thus, in India patients used CAM therapies since they were simply present [26]. Persons with cancer at Malawi thought the nearness of private health facilities serving traditional medicines made it available and considered it as a facilitator [21]. Some pregnant women in Ghana were also allowed in herbal medicine use by their midwives who perceived herbal medicine to be readily available [18]. However in Southeastern United States, nurses were concerned with the availability of materials needed to administer CAM in their hospitals which they considered as a barrier to CAM use [13]. The findings may communicate availability in diverse forms such as CAM therapies, human, material and infrastructural resources as a necessity to CAM therapies utilization at hospitals. Although the nurses in the current study and the midwives reported by Peprah et al. [18] seem to have focused on the availability of the herbal drug itself as a facilitator, it is important for health authorities in Ghana to consider availability in a much broader perspective.

The nurses narrated inadequate knowledge on herbal medicine as the main barrier to the clinical administration of herbal medicine and this has been reported in various countries including Ethiopia and Ghana [14, 18, 22, 47, 48]. As such, the nurses requested for further training on the herbal medicine [13, 14, 48] in order to be courageous in administering it. Interestingly, a number of studies have documented that knowledge on CAM therapies favors CAM utilization among health workers and patients [13, 47, 49]. For instance, some patients in the eastern part of Ghana who thought health care providers have inadequate knowledge in herbal drugs did not reveal their personal use of CAM to them. They considered the health professionals as strangers to such therapies [8]. Inadequate knowledge on CAM therapies as a common challenge might be considered as an overarching hindrance to administration or utilization of herbal medicine and other CAM interventions in different countries including Ghana. Nurses currently assisting or working at various herbal units in Ghana [19] might be an attempt to please leadership but this could be detrimental to patients due to their inadequate knowledge on herbal drugs [18].

The participants’ recommendation for specialist training in herbal medications perhaps might be a solution to the above challenge. The nurses assertion is partly consistent with [22] in which pharmacist demanded further empowerment of herbal medicine curriculum in Ethiopia. This presupposes a generally poor knowledge on herbal medicine among health professionals in Africa. In addition, the nurses’ recommendation might connote an over loaded curriculum in training health professionals; which might not be able to take further content on herbal medicines. Hence the need for a specialty training on herbal medicine to boost the knowledge of those who belief and opt for it.

Lack of promotion of herbal medicine was also reported as barrier by the participant and has been documented by [19, 24] in a private and public herbal clinic in Ghana. Probably, lack of publicity might be a common barrier to both public and private herbal units or clinics in Ghana. It is important for health authorities to advertise the herbal units since herbal medicine administration at hospitals in Ghana is a new phenomenon. According to [50] publicity creates familiarity and understanding of newly created institution, produce and services.

The nurses reportage of unclear integration as another barrier to the clinical administration of herbal medicine is supported by [19, 20]. In Ghana, Boateng et al. stated that some nurses thought there was no merger which was buttressed by some medical doctors in the same study [19]. Conversely, in the same study, other doctors perceived robust incorporation which was reinforced by the herbal doctors [19]. The nurses in the present study further recommended the need for collaboration between mainstream health professionals and staff of the herbal unit [51, 52]. Implying the absence of collaborative work at the facility which is also purported at the Kumasi South Hospital [19]. However, some medical and herbal doctors in the previous study echoed good communication and referral of patients between the conventional and non-conventional care systems [19]. This connotes conflicting thoughts on the state of collaborative work between herbal units and mainstream healthcare in Ghana. There is the need to bridge these divergent opinions especially as health workers work towards a common goal. Proper collaboration has been described as paramount to the effectiveness of the merger of CAM with conventional services [52] since it will promote sharing of ideas [51]. The contrary is reported to be dangerous to patients who combine CAM and orthodox medications [53].

The participants also revealed their ignorance of policies and protocols empowering them to serve herbal medicines in the future [13, 19]. As a result, the nurses felt not legally backed to serve herbal medicine. Boateng et al. in a similar finding at the Kumasi South Hospital described the merger of herbal medicine with conventional care as deceptive because of the absence of policies and protocols [19]. Thus, the absence of policies and protocols regarding herbal medicine administration in hospitals potentially leads to a feeling of insecurity among health professionals. This might possibly affect the level of commitment of nurses and other health professionals towards herbal medicine administration in hospitals. For instance, according to [54], health professionals should not opt for butterbur which is a herbal drug unless there are clearly written policies regarding it. In fact, there is the need for policies and protocols which must also be brought to the notice of conventional health workers to enhance herbal medicine administration in hospitals [51] recommended that hospitals offering nonconventional therapies must have official guidelines depicting the pathway to CAM utilization.

Another obstacle mentioned by the nurses is negative mindset of patients. According to the participants even if all health professionals agree to the administration of herbal drugs, negative mindset of patients will hinder the entire process. A study from Ghana by [24] submitted that some patients of a negative impression that herbal drugs have unpleasant taste refused to use the medication which was also considered as a barrier. It is essential for providers of herbal drugs to explore patients’ mindset since a favorable thought on CAM facilitates combination with conventional care provision [5].

Lack of National Health Insurance Scheme (NHIS) coverage was also identified as an impediment to hospital administration of herbal medicine which was also conveyed by [13, 24, 55] in the United States and Ghana. The current study and that of [24] which was carried out at a private herbal unit suggests lack of NHIS coverage for herbal medicine in both public and private herbal units in Ghana. A previous study at the Sekyere South District and Kumasi Metropolis of Ghana suggested that NHIS users were more potential for using traditional medicine from traditional medicine service providers whereas individuals without NHIS would be susceptible to the use of traditional medicine as a form of self-medication [56]. Similarly in Tanzania, uninsured hypertensive patients were also of a high tendency to use herbal medications because they had no funds for hospital care [11]. Meaning non coverage by NHIS might prevent patients from patronizing hospitals including the ones with herbal units in Africa; a situation if unattended to might potentially enhance the use of herbal medications outside hospitals. This might be dangerous because of the possibility of patients falling into the hands quack herbal practitioners. Policy makers should therefore consider integrating herbal medicine into NHIS to avert this anticipated danger. As narrated by [57] implementation of NHIS advances mainstream health care patronage.

The study did not include nurses from the other regional hospitals offering herbal services. The sample size of the study does not allow generalization of the findings.

## Conclusion

The study discovered detailed issues on the facilitators and barriers to the clinical administration of herbal medicine in Ghana as purported by the nurses. Facilitators to the clinical administration of herbal medicine included doctors’ prescription of the medication, patients’ ability to afford the medication, patients’ willingness to use the drug, beliefs of patient and availability of herbal medications. On the contrary perceived barriers such as inadequate knowledge on herbal medicine, insufficient publicity, indistinct integration and lack of collaboration between conventional and non-conventional care staff were recounted. Other barriers were ignorance on institutional policies empowering nurses to serve herbal medicines and lack of NHIS coverage of herbal drugs.

We therefore recommend an improvement in the content on herbal medicine in professional nursing training curriculum to enhance nurses’ knowledge. Furthermore, there must be proper collaboration between mainstream and nonconventional service providers. Policies on integration of herbal medicine into conventional care should be brought to the notice of nurses and other mainstream health workers. There is also a need to review and add herbal drugs to list of drugs covered by NHIS.

Future works may ascertain the receptiveness and level of preparedness of medical doctors to prescribe herbal medicine in the hospital. The state of policies on herbal medicine integration into mainstream health care in Ghana may also be explored.

## Data Availability

Data of the current study is available from the corresponding author upon considerable request.

## Abbreviations

CAM: Complementary and Alternative Medicine
NHIS: National Health Insurance Scheme
